# Injection Pultrusion of Glass-Reinforced Epoxy: Cure Kinetics, Rheology, and Force Analysis

**DOI:** 10.3390/polym16121642

**Published:** 2024-06-10

**Authors:** Fausto Tucci, Vitantonio Esperto, Germana Pasquino, Pierpaolo Carlone

**Affiliations:** 1 Department of Industrial Engineering, University of Salerno, 84084 Fisciano, Italy; vesperto@unisa.it (V.E.); pcarlone@unisa.it (P.C.); 2 Universitas Mercatorum, 00186 Rome, Italy; germana.pasquino@unimercatorum.it

**Keywords:** injection pultrusion, cure kinetics, thermoset rheology, force analysis

## Abstract

Pultrusion is a highly efficient continuous process to manufacture advanced fiber-reinforced composites. The injection pultrusion variant permits a higher control of the resin flow, enabling the manufacturing of a high reinforcement volume fraction. Moreover, it reduces the emission of volatile compounds that are dangerous for the operators and for the working environment. The present study proposes an experimental analysis of injection pultrusion in three different operative conditions. In particular, the activity focused on the effects of the temperature setup on the thermochemical and rheological behaviors of the resin system and on the interaction between the processed materials and the pultrusion die wall. The setup of the parameters was selected to evidence the behavior of the viscous interaction during the thermoset transition to the solid state, which is particularly challenging due to the localization of high adhesive forces related to the sharp increase in resin viscosity. Microscope observations of the cross-sections were performed to discuss the effects of the process parameters.

## 1. Introduction

Among advanced fiber-reinforced polymers, pultruded composites play a relevant role due to their high content of reinforcement and marked anisotropy [1]. These composites are shaped as constant cross-section profiles in thermoplastic [2,3] or thermoset [4] polymer reinforced by fibrous roving, fabrics, and mats [5]. The success of pultruded composites is related to their features and properties and to the highly advantageous manufacturing process. Indeed, pultrusion is the only continuous process to manufacture polymer matrix composites. This aspect promotes process automation, repeatability, productivity, and energy and cost reduction [6]. The pultrusion of the thermoset is based on the online impregnation of the fibrous reinforcement and the successive shaping and cure in a heated die. The conventional pultrusion process combines the resin and the reinforcement by diving the dry fibers inside an open bath of resin [7,8]. This procedure is extremely simple but presents several remarkable drawbacks: emission of toxic volatile compounds, uncontrolled resin flow, waste of resin, and issues related to the resin pot life. The injection pultrusion process is a variant of conventional pultrusion developed to overcome these limitations. In this case, the thermoset is injected through the dry reinforcement in a closed injection chamber [9].

Short processing time is the main feature of pultrusion. The raw materials are transformed into the final product in the die-crossing time, typically ranging between 1 and 10 min. In this time interval, the polymerization reaction should be activated and completed. If the glass transition occurs downstream of the die outlet, marked shape distortions are highly likely to occur [10]. The polymerization reaction is activated through the thermal energy provided by the electrical heating platens. Nevertheless, a premature polymerization of the thermoset could give place to incomplete reinforcement impregnation. Such an issue is particularly relevant in the case of injection pultrusion. The polymer–reinforcement combination is efficiently described by Darcy’s law, which relates the flow velocity of fluids through a porous medium (the fibrous reinforcement) to the fluid viscosity, the permeability of the medium, and the pressure gradient [11,12,13,14]. The viscosity of the thermoset systems sharply increases once the polymerization is activated [15]. Therefore, the heating energy provided and the pulling speed dictating the reaction time must be calibrated in order to promote a complete impregnation of the reinforcement and, on the other hand, to achieve a satisfactory polymerization and glass transition within the die.

The thermoset resin reaction and the parameters’ setting also affect the resistance opposed by the pultruded materials to the pulling force. The scientific literature analyzes the resistant forces and distinguishes them into collimation force, bulk compaction, viscous drag, and solid friction [16,17,18]. The collimation force is related to the terms of resistance from the creel rack to the injection chamber inlet. They are related to the pre-tension of the reinforcement and the contact with the guiding and preforming plates. This term is highly related to the peculiarities of each pultrusion line, and its analysis cannot be effectively generalized. Due to this, it is usually preferable to exclude this aspect from the force analysis of the process [19]. The bulk compaction is due to the constraining of a high amount of fibers in the narrow cavity of the die, and it increases depending on the final volume fraction of fiber [16]. Viscous drag is related to resin rheology. At the activation of the curing reaction, the resin viscosity starts increasing. Depending on the kinetic and temperature conditions, the viscosity increase can be highly sharp [20]. Immediately upstream of the solid transition zone, the viscosity is extremely high, and this gives place to localized peaks of the viscous drag, which often represent the most relevant resistance to the pulling action [21]. Finally, after the solid transition, the interaction between the die and the advancing composite is based on solid friction. The intensity of the resistance is related to the thermal expansion and the chemical shrinkage of the pultruded composite [22]. The pulling force is responsible for residual tensions in the composite profile which can induce matrix cracks and internal defects.

The present document describes an experimental work to analyze the injection pultrusion processes for the production of glass-roving-reinforced epoxy. In particular, composite profiles have been produced with different process parameters to evaluate the influence of temperature and cure evolution on the pulling force, local interaction between processed materials and die walls, and the internal quality of the produced composite. The different data collected have been cross analyzed to quantitatively assess the mechanisms characterizing the injection pultrusion. The cross-sections of the pultruded composites have been observed by optical microscope to assess the continuity of the matrix and detect the eventual presence of defects and cracks.

## 2. Materials and Methods

**Figure 1:**
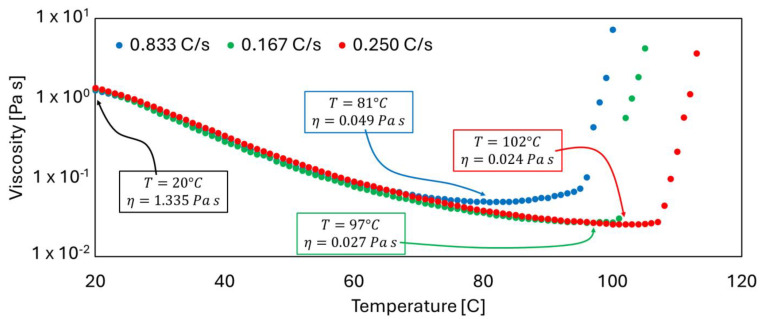
Rheological behavior of the epoxy resin system.

A laboratory-scale injection pultrusion die was adopted to pultrude E-glass-roving-reinforced thermoset. The composites pultruded have rectangular cross-sections 12 mm wide and 5 mm thick. The resin system was obtained by mixing Elan-Tech EC114 epoxy and Elan-Tec W132 hardener (Elantas, Wesel, Germany). The mass ratio of hardener and epoxy adopted was 37:100, as per the producer’s suggestion. The ingredients were mixed under vacuum conditions at room temperature for 15 min in order to degas the resin. The curing cycle suggested by the producer for this resin system (4 h of curing at 90 °C) does not meet the requirements of a pultrusion process. However, the behavior of the resin at higher temperatures and with sharp heating ramps was characterized and evidenced characteristics suitable for pultrusion at temperatures up to 180 °C with sensibly shorter curing cycles. In particular, the resin system kinetics and rheology were previously characterized and are described, respectively, by Equations (1) and (2):

(1) $$R_{\alpha }\left(T,\alpha \right)=A_{0}\;exp\left(-\frac{E_{a}}{R\;T}\right)\;\alpha^{m}{\left(1-\alpha \right)}^{n}\;,$$

(2) $$\eta =A_{\eta }\;exp\left(\frac{B_{\eta }}{R\;T}+C_{\eta }\alpha +D_{\eta }\alpha^{2}\right)\;\;\;,$$

where $R_{\alpha }$ represents the cure reaction rate as a function of the temperature *T* and the degree of cure α, *A*_0_, and *A_η_* are pre-exponential coefficients, $E_{a}$ is the cure activation energy, *m* and *n* are cure reaction coefficients, *R* is the universal constant of ideal gases, *η* is the viscosity, and *B_η_*, *C_η_*, and *D_η_*, are the rheological coefficients. The rheological behavior measured during the experimental analyses of the resin system is illustrated in Figure 1.

At room conditions, the resin presents an average viscosity value of 1.335 Pa s. In Figure 1, it is possible to appreciate that by increasing the temperature from room conditions the resin system viscosity decreases up to about two orders of magnitude. At resin cure activation, the viscosity sharply increases. This commercial resin system has been developed to react in about 4 h at 90 °C. However, the study of the rheological behavior of this resin evidences that the reactivity depends on the thermal flow, and the process can be accelerated by increasing the heating rate and the processing temperature. Increasing the heating rate determines the decrease in the minimum viscosity and the increase in the reaction activation temperature. The numerical values of the kinetics and rheology coefficients calibrated experimentally in a previous work [21] are reported in Table 1.

**Figure 2:**
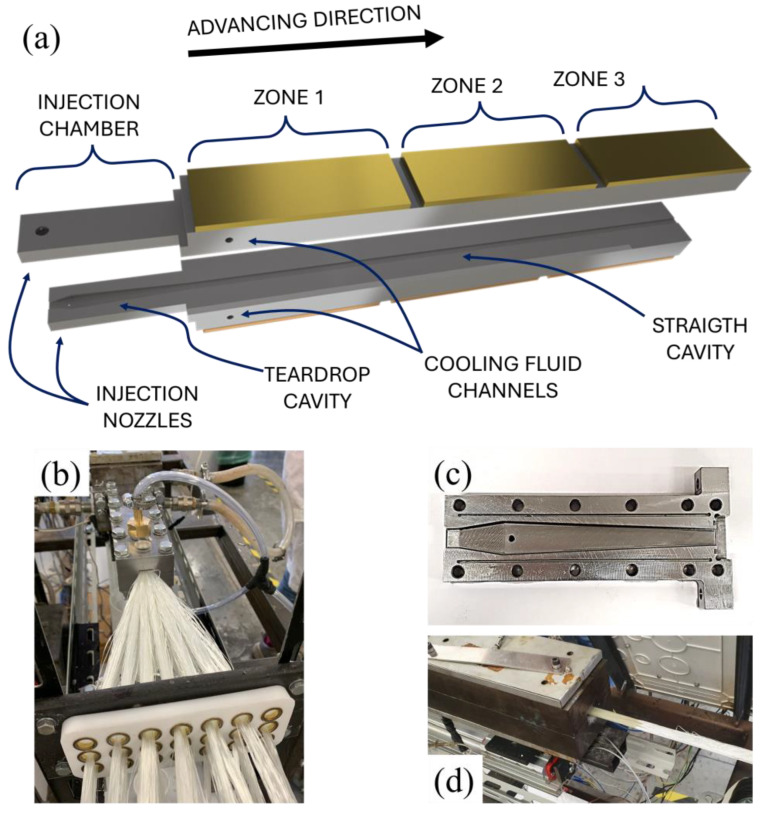
(**a**) Representation of the injection pultrusion line adopted; (**b**) glass fiber rovings guided towards the injection chamber inlet; (**c**) teardrop-shaped injection chamber cavity; (**d**) die outlet at the end of the initial transient.

The reinforcement consists of 37 rovings of E-glass fibers (tex. 2400), to achieve a volume fraction of fibers of 58%. The die adopted consists of a teardrop divergent–convergent injection chamber followed by a constant cross-section heating–curing die having a rectangular shape (12 mm × 5 mm). At the widest section of the injection chamber (34 mm × 12 mm), the pressurized resin system flows through two nozzles located over and below the advancing fibers, respectively. The divergent–convergent shape provides room to host resin and promotes the impregnation of the reinforcement. The initial divergent cavity is 40 mm long, while the convergent segment is 150 mm long. The injection chamber is mechanically connected with the 900 mm long straight die in order to guarantee the continuity of the cavity. The pultrusion line adopted is illustrated in Figure 2.

The resin system is injected at an absolute pressure of 1.5 atm through the advancing fibers within a teardrop-shaped injection chamber by two injection nozzles. In the photograph reported in Figure 2b, the reader can appreciate the diverging–converging teardrop shape of the injection chamber cavity. The pulling force applied by a caterpillar pulling system moves the materials with an advancing velocity of 180 mm/min. The pulling force has been kept constant in the different experiments to exclude velocity-related behavior and focus on the rheological interactions due to the cure and temperature evolution. Due to the same reason, the quantity of glass rovings pultruded, and the injection pressure was kept unaltered. A loading cell connected through an acquisition board to a laptop continuously measures the resistance to the pulling force. The die is heated by three pairs of electrical platens governed by PID controllers monitoring the surface temperature of the platens by means of thermocouples. The first couple of heaters are mounted on the external surface of the die, covering the die from a distance from the die inlet of 200 mm to 480 mm (zone 1), the second from 500 mm to 780 mm (zone 2), and the third from 800 mm to 1080 mm (zone 3). Each pair of heating platens can be autonomously controlled, permitting the realization of a thermal cycle to the processed materials. Two channels cross the heating die at 50 mm from the junction with the injection chamber. The water, used as a cooling fluid, absorbs the heat flow from the platens to prevent premature localized curing of the resin, which in turn can determine inhomogeneity in the composite or dramatical localized increase in the resistance to the pulling action. The experimental setup consists of three pultrusion runs with the temperature setpoints for the three couple of heaters reported in Table 2.

The heaters’ setpoints were chosen to guide a gradual growth of the temperature from the room conditions and provide the peak of heat flow in the central zone with the purpose of completing most of the curing process in a narrow portion close to the midway along with the heated die and gradually decrease the temperature to avoid excessive thermal shocks in the pultruded composite. Indeed, previous studies demonstrated the importance of completing most of the resin curing while the processed materials are well constrained into the die and the beneficial effects of this strategy on the shape distortion and the internal quality [23].

**Figure 3:**
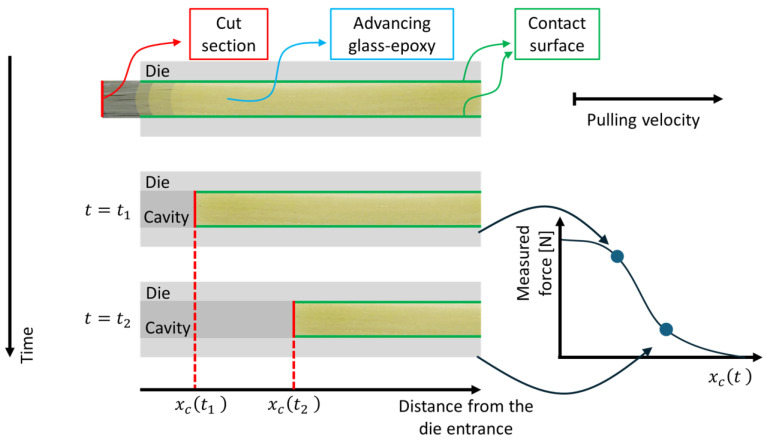
Schematic illustration of the fiber’s cutting method.

In Figure 2c the production of the first pultruded composite can be appreciated: the pulling of dry rovings is necessary for the start-up of the process. The dry fibers will be removed as scrap materials. This evidences that, at the start-up, an initial transient period must be considered. The stationarity of the process is assessed by observing the pulling force: once the measured resistance is stably oscillating within a limited range, the initial transient is considered over. When the process is stably stationary, a wire K-type thermocouple is fixed to the advancing dry fibers to evaluate the temperature profile adopting the traveling thermocouple method [21,24]. Finally, in order to evaluate the local resistance to the pulling action, the fibers’ cutting method is adopted, as illustrated in Figure 3.

The evaluation of the local resistance by the fibers’ cutting method. It is performed by cutting the fibers upstream of the die inlet and monitoring the evolution of the total load measured. The position of the cut section *x_c_* advances along with the die with a velocity equal to the pulling speed. While the cut section moves forward, the contact surface decreases; therefore, the total pulling force *Q* decreases accordingly. The local resistance *R_L_*(*x*) as a function of the longitudinal coordinate *x* is defined as the opposite of the derivative of the total pulling force, as described by Equation (3):

(3) $$R_{L}\left(x\right)=-\frac{dQ}{dx}\;\;\;.$$

Samples were collected from the pultruded profile and were mounted and polished to perform microscope observations of the cross-section. The observations were realized by using an optical microscope Nikon Eclipse L150 (Tokyo, Japan).

## 3. Results and Discussion

**Figure 4:**
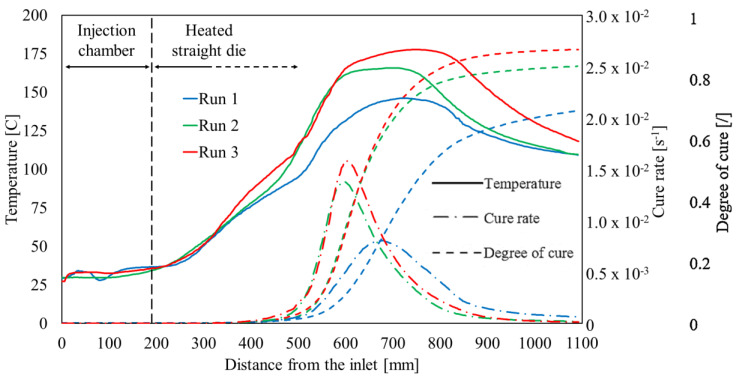
Temperature measured and kinetics behaviors evaluated in the three experimental runs.

The core temperatures measured with the traveling thermocouple method in the three pultrusion runs are illustrated in Figure 4, and they were compared with the cure rate and the degree of cure curves evaluated using Equation (1) and numerically integrating in time with the cure rates.

At the entrance, the measured temperature ranges between 27 °C and 29 °C, slightly higher than the laboratory conditions (about 23 °C) due to the proximity of the pultrusion line. Along with the first 230 mm of the cavity, the core temperature does not significantly increase since the fluid circulating through the cooling channels absorbs the heat flow emitted by the heating system. The resin system is considered completely uncured at the injection. Indeed, the reaction rate at room temperature is negligible for this thermoset polymer considering the time intervals from mixing to injection. Once the traveling thermocouple bulb overcomes the cooling channels, the temperature sensibly increases. The temperature curves almost overlapped for the first 350 mm. After that, even if the bulb is still moving through zone 1, the influence of the second couple of platens can be observed: the temperature of run 3 is higher than the ones of runs 2 and 1. At 400 mm from the entrance, the temperature values, ranging between 70 °C and 75 °C trigger the curing reaction, visible as activations of the cure rate curves. A further increase in the slope of the temperature curves can be observed between 450 and 500 mm from the entrance, where the processed materials approach the center of the line. The temperature values increase, driven by the high thermal power provided by the platens. On the other hand, the thermoset curing reaction is exothermal, and the emitted flux concurs with the temperature increase. This provokes a temperature crossover, consisting of materials’ temperatures being higher than the temperature of the heating system. For runs 1, 2, and 3, the maximum temperatures recorded in the three cases are, respectively, 146 °C, 166 °C, and 178 °C, sensibly higher than the platens setpoints in zone 2. The exothermal generation is proportional to the cure rate. The cure rate curves exhibit the typical bell-shaped trend with a peak in zone 2 of the die. The higher peak was reached in run 3, in which the temperature setpoint is higher. For run 1, the maximum value is clearly displaced towards the die outlet and is remarkably low, if compared to the other two cases. The temperature decreases along with zone 3. The outlet temperature measured is 110 °C for runs 1 and 2 and 118 °C for run 3. It is worth noting that, in run 3, the degree of cure evaluated at the end of the die is 0.92, while its results are equal to 0.86 and 0.78 for runs 2 and 1, respectively.

The pulling forces collected in stationary conditions in the three runs are, respectively, 174 ± 3.2 N, 136 ± 6.2 N, and 129 ± 2.2 N. The average value shows a remarkably higher value in run 1; nevertheless, the pulling force in run 2 exhibited sensibly higher variability. To deeply understand these behaviors, it is necessary to observe the local interactions between die and processed materials. According to the literature, the pulling force is given by the composition of bulk compaction, viscous drag, and solid friction [16]. The bulk compaction is evaluated as a function of the pressure field within the converging zone and the taper angle. Previous studies demonstrated that the pressure in the injection chamber depends on the pulling velocity, converging angle, injection pressure temperature, fiber volume fraction, and viscosity [15]. In this study, the velocity, the injection pressure, the number of fibers, and the geometry of the line were kept unaltered. Moreover, looking at the temperature and viscosity curves, low differences among the three runs can be observed; therefore, similar values of bulk compaction are expected in these tests. The viscous drag is active wherever the thermoset system is present at the liquid-viscous state, which means from the injection nozzles up to the cure transition. The viscous drag depends on the local viscosity value and on the velocity [21]. The solid friction is observable after the solidification of the matrix, which slides in contact with the cavity walls.

**Figure 5:**
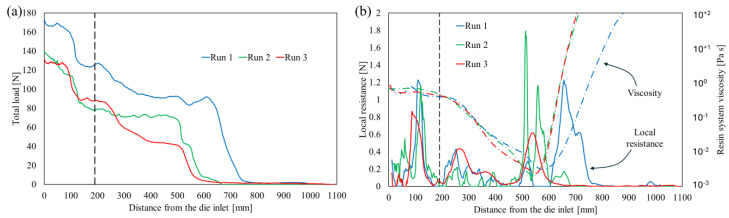
(**a**) Unloading curves; (**b**) local resistance curves and rheological evolution along with the die.

The unloading curves acquired during the three runs via the fibers’ cutting method are reported in Figure 5a, while the local resistance curves are compared to the rheological behavior in Figure 5b.

The unloading curves present qualitatively similar trends. The total load, initially close to the stationary value, does not sensibly decrease in the earliest portion of the cavity, while between 80 and 190 mm from the chamber entrance it presents a sharp decrease. Indeed, in this zone, the fibers are squeezed by the pulling action moving the reinforcement towards narrower cavity portions. This effect can be observed as a peak in the local resistance. This behavior has been described in the literature as bulk compaction, and it is expressed as follows [16]:

(4) $$F_{bulk}=\iint_{A_{t}}P\;\sin\theta \;dA_{t}\;\;\;,$$

where *P* represents the pressure, *θ* is the converging angle of the injection chamber cavity, and *A_t_* stands for the tapered area. Previous works [25,26] demonstrated that the pressure field in the converging chambers for injection pultrusion is variable along with the cavity, and it can increase by several tens of bars depending on the pulling speed, resin viscosity, injection pressure, and taper angle. Considering the shape of the injection chamber, the taper angle is equal to 1.3° in the thickness dimension and 4.1° in the width dimension. Within the converging zone, the bulk compaction is cumulated with the effects of the viscous drag. In order to analyze the value of the bulk compaction, it is necessary to evaluate and filter the viscous drag value from the local force measurement.

The viscous drag can be evaluated as a function of the pulling speed v, the local viscosity *η*, and the resin layer thickness *λ*, as described by Equation (5) [16]:

(5) $$F_{vis}=\frac{v}{\lambda }\iint_{A_{t}}\eta \left(\alpha ,T\right)\;\;dA_{v}\;\;\;,$$

therefore, with *l_x_*(*x*) being the perimetral line of the generic cross-section perpendicular to the advancing length, the derivative of the viscous drag with respect to the advancing direction coordinate *x*, representing the increase in the viscous interaction along with the cavity, can be evaluated as reported in Equation (6):

(6) $$\frac{dF_{vis}}{dx}=\frac{v}{\lambda }\int_{l_{x}}\eta \left(\alpha ,T\right)\;\;dl_{x}\;\;\;.$$

The value of the resin layer thickness, of course, is statistically variable in relation to the spatial distribution of the single fibers. Analytical expressions to determine the value of the thickness *λ* based on the fibers’ average radius *r_f_*, the fiber volume fraction *V_f_*, and the expected fiber packing were formulated. In the compacted scenario occurring in the case of these pultrusion runs and considering possible direct contact between fibers and cavity walls, the resin thickness can be expressed as follows [27]:

(7) $$\lambda =r_{f}\left(1-\frac{1}{2}\sqrt{\frac{\sqrt{3}\pi V_{f}}{2}}\right)\;\;\;.$$

**Figure 6:**
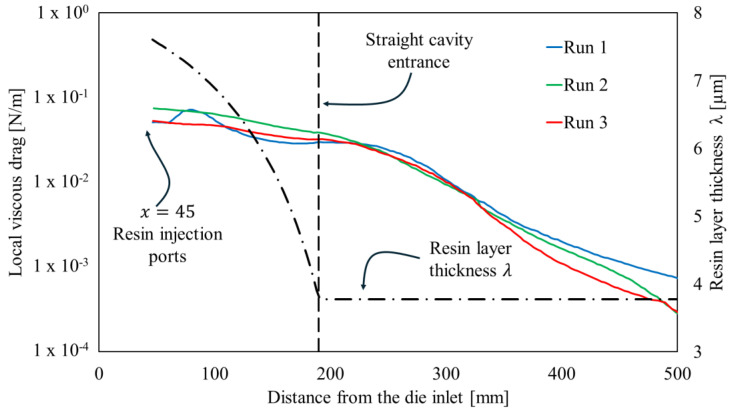
Local viscous drag and resin thickness layer along with the converging cavity.

Considering the resin rheology determining variations in the local viscous interaction and the decrease in the cross-section along with the convergent cavity, which implies a reduction in the resin thickness *λ*, the viscous drag can be expressed by the curves reported in Figure 6.

Filtering for the viscous drag reported in Figure 6 from the local resistance curves illustrated in Figure 5b, it is possible to detect and quantify the values’ bulk compaction peaks, which amount to 1.18 N/m at 110 mm from the die entrance, 1.09 N/m at 120 mm from the die entrance, and 0.82 N/m at 90 mm from the die entrance in run 1, run 2, and run 3, respectively. The temperatures measured during the three runs within the injection chamber were very similar since the cooling system mitigates the thermal differences. Nevertheless, due to the high sensitivity of the resin to the heat, the bulk compaction peaks are anticipated and less pronounced when the heating platens setpoints are higher.

**Figure 7:**
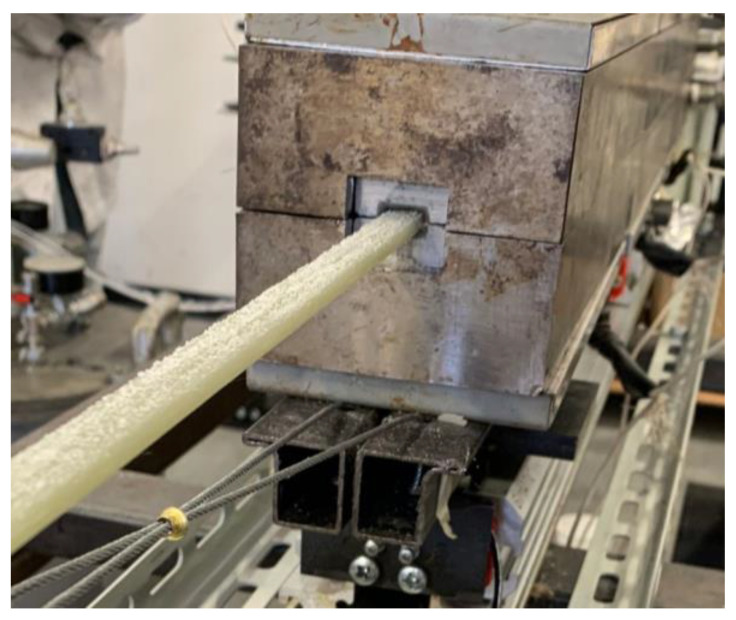
Close up on the pultruded profile during run 2.

Starting from the final zone of the injection chamber, the total load exhibits a low or negligible decrease along with the entirety of zone 1 and a large portion of zone 2. This is due to the deactivation of the bulk compaction in the straight cavity and the low resistance imposed by the viscous drag. Indeed, starting from 230 mm from the injection chamber entrance, the viscosity of the resin system starts sensibly decreasing, as shown in Figure 5b. Such a reduction is related to the increase in temperature previously observed, in agreement with Equation (2). In zone 2, in correspondence with the peak of the cure rate, the viscosity sharply increases, determining the drop in the total load measured and a peak in the local resistance. Finally, once the resin is solidified, the resistance to the pulling force appears to be negligible. This is related to the chemical shrinkage experienced by the thermoset polymers, which induces a reduction in the contact pressure between the processed materials and the cavity walls. In synthesis, all the runs present two locations of resistance concentration: the tapered injection chamber and the solid transition zone. The initial value of the total load in Figure 5a is the pulling force in stationary working conditions. The pulling force in run 1 is sensibly higher than the pulling force in runs 2 and 3 due to the delay in resin polymerization, which in turn determines a larger extension of the zone characterized by viscous drag, and the high and wide force peak observable during polymerization reaction in Figure 5b. The highest local resistance peak appears in zone 2 for runs 1 and 2 and in the injection chamber for run 3. The highest peak was observed for run 2. In this configuration, the reaction was sharper and highly localized. Probably, the high variability is directly related to this localized action, which determines stack and slip conditions, which in turn can be responsible for the visibly higher noise in the local resistance and for the highest variability of the total force in stationary conditions. This is also confirmed by the appearance of the pultruded profile surface at the die outlet reported in Figure 7.

It is possible to notice resin powder deposited on the pultruded surface. The powder was easily removed, and the composite surface was smooth as in the other two cases. The presence of the powder is the consequence of the stick and slip phenomenon, which provokes high localized tension in the solid transition zone and erodes the composite surface. On the other hand, the local resistance peak for run 1 in the transition zone is remarkably more extended: the reaction is slower and involves 200 mm of the die length, from 600 mm to about 800 mm. This peak is responsible for the highest value of the total load in the stationary process.

**Figure 8:**
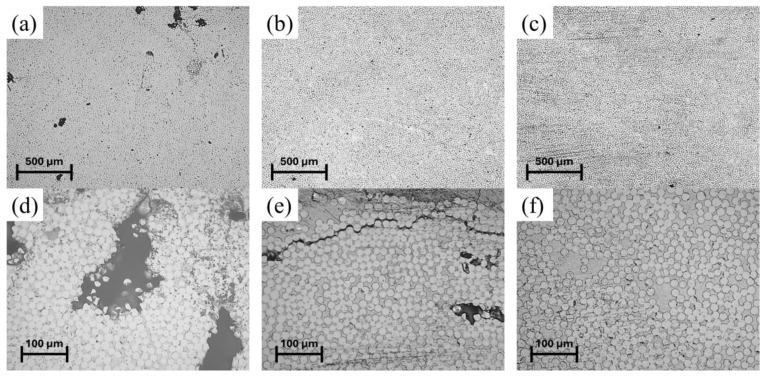
Microscope observation of the samples’ cross-section collected with different magnifications: (**a**) and (**d**) run 1; (**b**) and (**e**) run 2; (**c**) and (**f**) run 3.

The cross-section of the samples collected from the pultruded composites was analyzed by microscope observation, and some representative micrographs are reported in Figure 8.

All the samples observed generally exhibited good compaction and distribution of the reinforcement: no fiber-poor regions were observed. In the samples collected from the pultrusion produced in run 1, the presence of voids having dimensions of 100–500 µm was noted (Figure 8d). This can be related to the lower degree of curing reached at the end of the die and the consequent completion of the reaction without rigid constraints, which can provoke internal defects and porosities [10,23]. In the samples collected from the composite realized in run 2, the core of the pultruded profile appears solid and relatively free of visible voids. Nevertheless, in the zones close to the composite surface, large delamination cracks can be observed (Figure 8e). Such defects extend parallel to the exposed surfaces and could be the direct consequence of the local force increase during the solid transition in zone 2 of the die. The sample picked from run 3 presents good compaction and no visible extended defects.

## 4. Conclusions

This work focused on the effects of temperature cycles in the injection pultrusion of glass-reinforced epoxy. These effects were studied considering the thermoset system kinetics and rheology and adopting the force analysis to study the interaction between the materials processed and the die. The following was concluded from this study:

- In all the experiments conducted, two main loading zones were detected: in order of relevance, one corresponding to the resin transition and the consequent sharp increase in viscosity and another relative to the bulk compaction.
- Low processing temperature in the central zone of the die provokes a slower reaction and as a consequence an increase in the length of the reaction zone. Indeed, the viscosity curve during the reaction presents a remarkably lower slope. This implies higher pulling forces and incomplete curing of the resin, which in turn generates porosities and imperfections in the composite profile.
- The processing temperature also influenced the bulk compaction, despite the cooling fluid designed to thermally decouple the injection chamber from the die. Lower temperature corresponded to higher bulk compaction resistance and to a variation in the local resistance peak location. In the authors’ opinion, this aspect deserves further dedicated attention.
- An incorrect definition of the processing parameters can determine the risk of too-high localized actions. Such forces determine surface wear and transversal cracks at a few tens of micrometers below the surface, which can be highly dangerous for the structural stability of the composite.

The conducted research triggers new questions to be solved in future works dedicated to injection pultrusion processes. First of all, an accurate study of the mechanical behavior of pultrusion realized with different curing cycles will be of high interest to the scientific and industrial communities, in particular the interlaminar shear strength, which could be highly influenced by delamination cracks. Further investigations can be dedicated to the surface and dimensional analysis of the composite to quantify the possible wear and roughness, as well as the geometrical stability of the profiles. An exhaustive and validated numerical model of the local resistance to the pulling force and its effects on the internal and surface quality of the pultruded composite should be defined. The aforementioned studies would enable the development of diagnosis strategies based on the stationary pulling force signal, analyzing, in particular, its average value and variability.

## Figures and Tables

**Table 1:** Numerical values of the kinetics and rheology coefficients.

Coefficient [Unit]	Value
$A_{0}\;\;\;\left[s^{-1}\right]$	4.18 × 10^4^
$E_{a}\;\;\;\left[J\;\;mol^{-1}\right]$	4.97 × 10^4^
m [/]	4.14 × 10^−1^
n [/]	1.87 × 10^0^
$A_{\eta }\;\;\;\left[Pa\;\;s\right]$	1.22 × 10^−11^
$B_{\eta }\;\;\;\left[J\;\;mol^{-1}\right]$	6.22 × 10^4^
$C_{\eta }\;\;\;\left[/\right]$	9.75 × 10^0^
$D_{\eta }\;\;\;\left[/\right]$	1.25 × 10^1^

**Table 2:** Heating platens settings.

Pultrusion Run	Heating Platens Temperature Setpoint [C]		
	Zone 1	Zone 2	Zone 3
Run 1	90	140	100
Run 2	90	155	100
Run 3	90	170	100

## Data Availability

The original contributions presented in the study are included in the article, further inquiries can be directed to the corresponding authors.
